# AI-integrated manufacturing of advanced materials for energy storage, catalysis, and environmental applications

**DOI:** 10.3389/fchem.2026.1864044

**Published:** 2026-07-03

**Authors:** Sai Kumar Punna, Suvarshitha Pusuluru, Madhumita Ravikumar, Farid Menaa

**Affiliations:** 1 Department of Materials Science and Engineering, University of Houston, Houston, TX, United States; 2 Department of Engineering Data Science, University of Houston, Milwaukee, TX, United States; 3 Department of Bioengineering, Saveetha School of Engineering, Saveetha Institute of Medical and Technical, Chennai, India; 4 Department of Medicine, Medical College of Wisconsin, Milwaukee, WI, United States; 5 Department of Biomedical and Environmental Engineering (BEE) California Innovations Corporation (CIC), San Diego, CA, United States

**Keywords:** artificial intelligence, catalytic materials, CO_2_ capture, electrocatalysis, energy sustainability, machine learning, metal-organic framework

## Abstract

The incorporation of artificial intelligence (AI) into energy systems has become a transformative strategy for tackling global energy related challenges, particularly energy vulnerability (EVI). This work examines how AI contributes to mitigating EVI by evaluating its influence across several dimensions, including energy availability, operational efficiency, consumption patterns, renewable energy integration, and overall energy security. Based on insights derived from machine learning (ML) enabled developments in catalytic materials and CO_2_ capture technologies, this study demonstrates how data-centric approaches expedite material discovery, refine energy processes, and strengthen system resilience. ML methodologies, including artificial neural networks (ANN), support vector regression (SVR), and ensemble learning techniques, exhibit strong predictive performance in estimating activation energies, adsorption properties, and catalytic efficiencies. These methods substantially decrease reliance on computationally intensive density functional theory (DFT) simulations, thereby enabling rapid identification of high-performance catalyst. Moreover, ML-assisted framework supports the detection of active catalytic sites, these optimization of electrocatalytic processes, and the design of materials for hydrogen evolution, CO_2_ reduction, and ammonia synthesis. Simultaneously, ML applications in CO_2_ capture systems particularly in metal-organic frameworks (MOFs) facilitate high throughput screening and predictive evaluation of adsorption capacity and structural behaviour. Through the application of quantitative structure-property relationships and feature importance analyses, ML models identify key variables governing CO_2_ capture performance, thus lowering computational demands and accelerating material development. The study highlights the rise of integrated, closed-loop systems that combine ML, theoretically modelling, and automated experimentation to streamline catalyst development and carbon capture process. Collectively, the results indicate that AI-driven methodologies substantially improve the efficiency, sustainability, and scalability of advanced energy technologies. These developments not only help mitigate energy vulnerability but also promote the global shift toward low-carbon, resilient energy systems.

## Highlights

- Machine learning expedites catalyst discovery through accurate prediction of critical reaction and adsorption parameters.
- AI-based models reduce reliance on computationally intensive DFT simulations.
- ML improves the optimization of electrocatalytic processes for hydrogen production and CO_2_ conversion
- Data-driven screening improves MOF-based CO_2_ capture performance and material selection.
- AI-based approaches enable faster and more efficient development of advanced energy materials.

## 1 Introduction

### 1.1 Machine learning methods to assist energy system optimization

Machine learning-based strategies are increasingly utilized to improve the optimization of contemporary energy systems. Distributed energy architecture offers a flexible platform for incorporating intermittent renewable sources such as solar photovoltaic (SPV) and wind power. Within this framework, concepts including virtual power plants, smart microgrids, energy hubs, and integrated multi-energy systems have emerged, facilitating the integration of non-dispatched resources while reducing negative effects on grid stability (Asmus, 2010; Chicco and Mancarella, 2009). By integrating components with varying operational features, distributed systems can ensure reliable energy delivery even during intervals of low renewable generation. Nevertheless, this diversity introduces complex interactions in energy flows, thereby requiring advanced dispatch optimization techniques (Ackermann et al., 2001). A central challenge in energy system design is the concurrent optimization of system configuration and operational control. Optimization models must simultaneously consider component selection and dynamic dispatch decisions under fluctuating demand conditions and variable renewable energy availability. This complexity is further amplified by uncertainties in load demand, renewable resource potential, and market dynamics (Fathima and Palanisamy, 2015; Mavromatidis et al., 2018; Wang et al., 2018). Bi-level optimization methods have been widely employed to address this challenge, where system design is optimized at the upper level and operational strategies are resolved at the lower level (Agustín and López, 2009; Banos et al., 2011)]. Within such frameworks, dispatch optimization is conducted to estimate operational cost based on time-dependent variations in demand, renewable supply, and grid interactions (Evins, 2015; Fazlollahi et al., 2012; Fazlollahi et al., 2015). Despite their effectiveness, these methods are computationally intensive, particularly when incorporating detailed engineering models and high-resolution temporal datasets. For example, large-scale implementations involving annual (8760-h) simulations combined with mixed-integer linear programming (MILP) may require several days of computation, even when parallel computing is employed (Evins, 2015). To reduce computational demands, surrogate (meta-) modelling approaches have attracted considerable interest. These models approximate the behaviour of detailed engineering simulations, thereby decreasing the computational effort required for objective function evaluation (Narayan and Ponnambalam, 2017). Data-driven approaches such as artificial neural networks support vector machines (SVM) and random forest algorithms have demonstrated strong capability in representing complex system relationships. Studies indicate that surrogate models can reduce computational time by more than two orders of magnitude while maintain prediction accuracies above 90% (Evins, 2013). As a result, these models have been widely adopted across multiple engineering fields, including civil, automotive, aerospace, manufacturing, and building energy simulations (Chen and Yang, 2018). Within energy systems, surrogate models have predominantly been applied at the component level. Representative applications include optimization of proton exchange membrane (PEM) fuel cells (Cheng et al., 2013; Miao et al., 2011), wave energy converts (Halder et al., 2017; Halder and Samad, 2017; Thomsen et al., 2018), and turbomachinery systems such as axial turbines used in liquefied natural gas processes (Kadhim and Rona, 2018a; Kadhim and Rona, 2018b). In these cases, computationally expensive models-such as computational fluid dynamics (CFD) are replaced with surrogate approximations to accelerate design optimization. However, extending these methodologies from individual components to integrated energy systems introduces additional complexities due to the larger number of interacting variables and constraints. System-level applications of surrogate models remain limited. Some investigations have employed surrogate-based optimization for simplified energy system designs, such as optimizing surrogate-based optimization for simplified energy system designs, such as optimizing collector area and storage capacity in solar thermal systems (Bornatico et al., 2013) or approximating complex process models in renewable ammonia production systems (Sanchez and Martín, 2018). However, these approaches often overlook operational dispatch strategies, focusing primarily on static design parameters. Incorporating dispatch within surrogate models significantly increases problem dimensionality and complexity, making accurate approximations more challenging. Consequently, exclusive reliance on surrogate models may result in suboptimal solutions due to approximation inaccuracies in highly nonlinear and dynamic systems. This highlights the necessity for hybrid optimization frameworks that combine surrogate models with high-fidelity engineering models (AEMs), thereby achieving a balance between computational efficiency and accuracy. Furthermore, traditional bi-level optimization approaches frequently require complete re-optimization when key inputs such as renewable availability, demand profiles, or techno-economic factors-change, leading to substantial computational overhead. This constraint limits their applicability in large-scale or regional planning contexts where multiple distributed systems must be optimized simultaneously. Addressing these challenges remain a critical area for advancing research in energy system optimization.

## 2 Overview of the computational framework

Energy system design is typically formulated as a simulation-driven optimization problem in which system performance is assessed under time-dependent conditions, including renewable resource availability, energy demand, and grid interactions. The relationship between decision variables (design parameters) and objective functions is established through time-series simulations, generally spanning 8790 hourly intervals (24 × 365) or a reduced set of representative periods. This process can be interpreted within the framework of a Markov Decision Process (MDP), where system states evolve dynamically over time. Since objective functions depend on both system configuration and operational (dispatch) strategies, their simultaneous optimization substantially increases computational complexity, often resulting in execution times extending over several days. This section presents the computational methodologies utilized, including conventional techniques and a proposed framework incorporating supervised learning and transfer learning approaches. Section 2.1 outlines the role of actual engineering models (AEMs), while Sections 2.2 and 2.3 introduce advanced computational strategies designed to reduce computational cost. Section 2.4 further explores that extension of this framework to diverse geographical regions through transfer learning.

### 2.1 Actual engineering models (AEMs)

The operational strategy of an energy system including the selection and scheduling of dispatchable generation units, storage systems, and grid interactions has a direct impact on optimal system sizing. Therefore, system operation must be evaluated on an hourly basis to accurately capture variations in renewable generation, demand patterns, and grid conditions. Two primary approaches derived from dynamic programming theory are commonly used for this purpose (Perera et al., 2017; Piacentino and Cardona, 2008). The first approach is based on the value function method, in which the optimal operational strategy is determined at each time step. This method, in which the optimal operational strategy is determined at each time step. This method is well-suited for complex multi-energy (poly-generation) systems but is computationally demanding due to high dimensionality of the state space. The second approach utilizes a policy function method, where an optimal operational policy is assumed and optimized simultaneously with system design variables. Although less computationally intensive, this method still requires full temporal simulation across all time considerable computational requirements. In the present study, the policy function-based approach is adopted within the AEM framework. The AEM fulfils two primary roles: (i) generating high-quality datasets for training surrogate models, and (ii) enabling the mapping of decision variables to objective functions within the proposed hybrid optimization framework. A comprehensive description of the AEM is provided in Section 3.

### 2.2 Development of surrogate models supervised learning

To overcome the computational limitations associated with AEM-based optimization, surrogate models are employed as efficient approximation of system behaviour. These models, typically developed using machine learning techniques such as neural networks, support vector machines (SVM), and other regression-based approaches, establish functional relationships between input variables (decision parameters) and output variable (objective functions) without explicitly solving the underlying physical models. Although surrogate models provide substantial computational benefits, they interpretability. Instead, they function as complementary tools that reduce the computational burden of repeated time-series simulations. In energy system optimization, surrogate models are mainly utilized to approximate the mapping between decision space and objective space, thereby accelerating the evaluation of candidate solutions. The development of a surrogate-assisted optimization framework generally involves three main stages:

### 2.3 Dataset Generation

A comprehensive dataset is produced using the AEM by simulating system performance across various design and operational scenarios. This dataset serves as the foundation for training and validation.

### 2.4 Model training

Supervised learning techniques are applied to train the surrogate model, enabling it to capture nonlinear relationships between input variables and system performance metrics. Proper model selection and validation are essential to ensure predictive accuracy and generalization capability.

### 2.5 Optimization

The trained surrogate model is integrated with an optimization algorithm (e.g., heuristic or evolutionary techniques) to determine optimal system configurations. Since surrogate evaluations are computationally inexpensive, the overall optimization process becomes significantly more efficient.

Although surrogate models effectively reduce computational time, they primarily approximate system responses and do not inherently perform optimization. Therefore, the integration of appropriate optimization technique remains necessary to obtain optimal design solutions.

### 2.6 Hybrid optimization framework integration surrogate models and AEMs

Actual engineering models (AEMs) provide high-fidelity evaluations of objective functions and are therefore more accurate than surrogate models. However, their application in large-scale optimization is computationally expensive, especially when repeated evaluations are required. Conversely, achieving high predictive performance with surrogate models require extensive training datasets, which themselves demand considerable computational effort to generate using AEMs. To address this trade-off, a hybrid optimization algorithm (HOA) is proposed, combining the computational efficiency of surrogate models with the accuracy of AEMs. In this framework, the surrogate model is initially coupled with a optimizing algorithm to rapidly explore the solution space and generate a preliminary Pareto front. Due to the low computational cost of surrogate evaluations, this stage enables efficient convergence toward near-optimal solution regions. Subsequently, the solutions obtained from the surrogate-assisted phase are used as initial candidates for a refinement stage employing AEMs. During this phase, decision variables are re-evaluated through high-fidelity simulations, enabling more precise mapping between decision space and objective space. The AEM-based refinement ensures that the final Pareto front accuracy reflects system behaviour while maintaining computational efficiency. Overall, the HOA capitalizes on the strength of both approaches: rapid convergence facilitated by surrogate models and high accuracy ensured through AEM-based validation and refinement.

### 2.7 Transfer learning for model generalization

A significant limitation of conventional methodologies is the requirement to restart the entire optimization process when applying the analysis to a new location, resulting in substantial computational overhead-particularly in large-scale regional or national studies involving multiple distributed energy systems. To address this limitation, transfer learning techniques are implemented to improve the adaptability of surrogate model. Unlike traditional training approaches, transfer learning allows knowledge gained from a source domain (initial location) to be reused in a target domain (new location), thereby enhancing model performance with reduced training effort. This strategy significantly lowers the need for large, labelled datasets and reduces the computational cost associated with retaining. In practice, a surrogate model developed for an initial location is adapted to a new location by retaining it using a smaller dataset generated from AEM simulations under the new conditions. The data generation process can be further accelerated using high-performance computing techniques, such as GUP-based parallelization. By incorporating this limited yet representative dataset, the surrogate model is fine-tuned to capture the characteristics of the new environment. The transfer learning enhanced surrogate model is subsequently integrated into the optimization framework, enabling efficient and scalable energy system design across multiple locations. This approach not only reduces computational time but also improves the feasibility of applying optimization models in large-scale planning scenarios where numerous distributed systems must be analysed simultaneously.

## 3 Hybrid surrogate modelling for energy system representation

A variety of machine learning methodologies, including support vector machines (SVM), artificial neural networks (ANN), and linear regression (LR), can be utilized for the development of surrogate models. Among these approaches, ANNs have shown superior performance across a wide range of applications, particularly considering recent progress in deep learning techniques. Consequently, ANN-based architectures are employed in this study to construct surrogate representation of the energy system. An ANN is composed of interconnected neurons arranged in multiple layers, where each neuron applies a nonlinear transformation to the input data. Identifying an optimal network architecture remains a challenging task due to the extensive design space defined by the number of layers and neurons within each layer. Model training is conducted using the Levenberg-Marquardt backpropagation algorithm, which follows a damped least-squares optimization approach. The mean squared error (MES) is used as the lost function, and no regularization is applied during training. The samples for training, 192,000 samples for validation, and 640,000 samples for testing. Following this, transfer learning is implemented to adapt the trained model to new scenarios. During this stage, the pretrained network is fine-tuned using a considerably smaller dataset, comprising of 7,000 training samples, 3,000 validation samples, and 10,000 testing samples, while retaining the same hyperparameter settings as used in the initial training phase. Model performance is assessed using mean absolute error (MAE). The results demonstrated that increasing both the depth and width of the network generally leads to improvement in predictive accuracy. For example, progressively increasing the number of layers results in a reduction in prediction error, as observed across architecture AR1 through AR6. However, deeper architecture also led to increased computational cost during both the training and inference stages. To achieve a balance between accuracy and computational efficiency, widening strategies are investigated by increasing the number of neurons within each layer. Architectures such as AR7 and AR8 exhibit significant reduction in prediction error with relatively lower computational expense. Although further increase in both depth and width (e.g., AR9) provide only marginal improvements in accuracy, the corresponding rise in computational complexity makes such configurations less practical. Therefore, architecture AR8-characterised by a moderately wide and shallow structure is selected as the optimal surrogate model in this study, offering an effective trade-off between predictive accuracy and computational efficiency. Several quantitative studies demonstrate the practical advantages of AI/ML methods in energy system and energy material applications. In distributed energy system optimization, an ANN based surrogate model combined with a hybrid optimization algorithm reduced computational time by up to 84% and achieved Pareto-optimal solutions approximately 17 times faster than the actual engineering model. This illustrates how surrogate assisted optimization can accelerate complex technoeconomic energy system design while maintaining acceptable accuracy. In advanced energy materials discovery, ML has also shown significant acceleration potential. Gaussian-process based ML models have been used to predict gas separation behavior for more than 11,000 untested homopolymers using permeability data from approximately 700 polymers, thereby expanding the screening space far beyond what is practical through conventional experiments alone. Similarly, ML based generative models have been applied to generate porous electrode microstructures, reducing the computational burden of electrochemical simulations.

Although many AI/ML studies focus on prediction accuracy, the physical relevance of predicted descriptors is equally important, in battery systems, performance metrics such as capacity retention, Coulombic efficiency, impedance growth, ionic conductivity, redox potential, and degradation rate are not isolated numerical outputs; rather, they are linked dynamic physicochemical processes occurring across multiple length scales. Electrolyte descriptors such as coordination energy, oxidation/reduction potential, dielectric constant, viscosity, and solvation structure can influence transport, interfacial charge transfer, and the stability of the solid electrolyte interphase (SEI). NL studies on electrolyte materials have shown that coordination energy and redox-potential descriptors can guide the design of advanced electrolyte systems, because these descriptors are directly related to ion transfer and interfacial reactions. Recent operando studies show that SEI formation and electrolyte strain are spatially heterogenous and evolve dynamically during cycling. Therefore, AI-predicted descriptors should be interpreted in relation to these physical processes. For instance, descriptors associated with electrolyte reduction potential and additive chemistry may be linked to SEI nucleation, growth, thickness, and chemical stability. Similarly, cathode composition, particle size, lattice parameters, elastic properties, and lithium diffusion barriers may be connected to strain heterogeneity, phase transformation, cracking, and capacity fading. Thus, AI models can become more physically interpretable when feature importance, SHAP analysis, sensitivity analysis, or physics-informed learning is used to connect model outputs with mechanistic phenomena such as SEI evolution, local stress development, interfacial instability, and ion-transport limitations. A useful future direction is to integrate operando characterization data, such as optical imaging, X-ray microscopy, diffraction, spectroscopy, and electrochemical impendence measurements, with ML models. This would allow AI predicted performance metrics to be validated against real-time physical changes in electrode and interfaces. Such integration can move AI from purely data driven prediction toward mechanism aware materials discovery and battery optimization.

### 3.1 Data availability and quality

AI models require reliable, diverse, and machine-readable datasets. However, energy materials data are often scattered across publications, reported in inconsistent formats, or limited to successful experiments. Failed or negative results are rarely reported, creating bias in the training data. Liu et al. emphasized that data scarcity and lack of standardization remain major barriers in data driven energy materials research. Future research should focus on open databases, standardized reporting formats, inclusion of failed experiments, automated data extraction, and integration of experimental, computational, and industrial datasets.

### 3.2 Model interpretability

Many AI models, especially deep neural networks, behave as black-box predictors. This limits user trust and makes it difficult to connect predictions to physical mechanisms. Future studies should incorporate explainable AI tools, feature-importance analysis, SHAP values, symbolic regression, and physics-informed ML. In battery research, model interpretation should connect predicted outputs to electrochemical mechanisms such as SEI growth, ion diffusion, electrode strain, phase transformation, and degradation pathways.

### 3.3 Scalability and transferability

Models trained in one material class, battery chemistry, manufacturing system, or energy system may not perform well under new conditions. Perera et al. showed that transfer learning can adapt surrogate models to new scenarios involving changes in solar potential, wind speed, and energy demand, enabling faster optimization under varying conditions. Future work should emphasize transfer learning, domain adaptation, federated learning, and hybrid physics-AI models to improve scalability across materials, devices, and operating environments.

### 3.4 Real world implementation

A major gap remains between algorithm development and industrial validation. In energy-efficient manufacturing, only a limited number of AI methodologies have been validated in real-world environments, and challenges remain in online implementation, dynamic adaptation, privacy-preserving data collection, and human-AI collaboration. Future research should include pilot-scale demonstrations, digital twins, closed-loop optimization, and autonomous laboratories to validate AI predictions under realistic operating conditions.

### 3.5 Optimization framework

Optimization methodologies are applied at two separate stages (i) the training of the surrogate model and (ii) the optimization of the energy system design. During the training phase, stochastic gradient descent (SGD) is employed to iteratively minimize the loss functions and update the network parameters. For system-level optimization, both the AEM and the surrogate model act as mapping between decision variables (design and operational parameters) and objective functions. Within the AEM framework, this mapping is achieved through time-series simulations that incorporates both system configuration and dispatch policy variables. In contrast, the surrogate model utilizes a fully connected feedforward ANN to approximate this relationship. An appropriate optimization algorithm is required to determine optimal solutions regardless of the modelling approach used. Conventional techniques such as linear programming (LP) and mixed-integer linear programming (MILP)have been widely adopted in energy system design due to their computational efficiency and ability to handle structured constraints. However, surrogate models based on neural networks typically produce nonlinear and non-convex objective functions, thereby limiting the applicability of these traditional optimization methods.

Accordingly, the optimization strategy adopted in this work must be capable of effectively interfacing with both high-fidelity AEMs and nonlinear surrogate models. This requirement necessitates the use of advanced optimization techniques beyond conventional deterministic or purely heuristic approaches, ensuring reliable convergence toward optimal or near-optimal solutions within a computationally efficient framework. The surrogate model provides a direct mapping between the decision vector and the objective space, enabling rapid estimation of objective function values and constraint violations. These outputs are then passed to the evaluation module, where objective values are assessed and both the solution population and archive are iteratively updated. Similarly, when the actual engineering model (AEM) is utilized, an analogous evaluation structure is followed; however, the computational pathway differs. In this case decision vector is processed through a detailed time-series simulation that incorporates hourly variations in wind speed, solar irradiance, and energy demand, as described in Section 3. The resulting objective function values and constraint violations are subsequently transmitted to the evaluation stage for updating the population and Pareto archive.

### 3.6 Hybrid optimization algorithm (HOA) integrating AEM and surrogate models

The optimization workflow using either the surrogate model or the AEM independently, direct integration of these two approaches requires a modified computational structure. The surrogate model offers high computational efficiency but may exhibit reduced accuracy in predicting objective functions and constraint satisfaction under certain conditions. In contrast, the AEM provides more accurate, high-fidelity results but at the cost of significantly increased computational time. To capitalize on the advantages of both methods, a hybrid optimization algorithm (HOA) is introduced. In the initial phase, the surrogate model is combined with the optimization algorithm to rapidly explore the decision space and generated an approximate Pareto-optimal solution set. This stage benefits from the low computational cost of surrogate evaluations, enabling efficient convergence toward promising regions of the solution space. The resulting solution population and Pareto archive are then transferred to a subsequent stage, where their objective function values and constraint violation are re-evaluated using the AEM. This validation step is critical for correcting any inaccuracies introduced by the surrogate approximation. Following re-evaluation, the dominance relationships among the solutions are reassessed, and both the solutions population and archive are updated accordingly. The refined solution set is then used as an informed starting point for the final optimization stage, in which the AEM is fully incorporated into the optimization loop. This allows for further refinement and convergence of the Pareto front with high accuracy. Overall, the HOA framework integrates the rapid exploratory capabilities of surrogate models with the precision of AEM-based evaluation, resulting in an optimization process that is both computationally efficient and reliable.

### 3.7 Artificial intelligence and machine learning in energy systems

**FIGURE 1:**
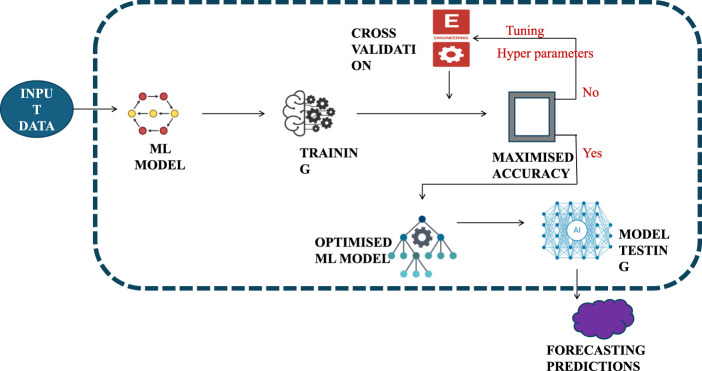
Typical workflow of an ML model.

Economic development and improvements in societal wellbeing are closely associated with rising energy demand. However, meeting this demand solely through increased energy production is neither sustainable nor equitable. From the perspective of energy justice, the ongoing depletion of finite resource raises significant concerns regarding intergenerational equity (Asmus, 2010). While measures such as retrofitting existing infrastructure and improving system efficiency though approaches like energy cascading and system integration can help reduce energy consumption, efficiency improvement alone may not provide a complete solution. In many situations, systems can be dynamically operated at partial loads or temporarily shut down, indicating that adaptive and responsive operational strategies may yield greater long-term benefits. Within this context, artificial intelligence (AI) and machine learning (ML) have emerged as powerful tools for enhancing the operation and management of energy systems (Figure 1). The concept of artificial intelligence, first introduced by John McCarthy in 1954 (Chicco and Mancarella, 2009), describes the ability of machines to emulate human cognitive processes such as learning, reasoning, and problem-solving (Ackermann et al., 2001). Over time, AI has evolved into a broad and rapidly expanding discipline, with applications spanning sectors including healthcare, finance, manufacturing, agriculture, and security (Fathima and Palanisamy, 2015; Mavromatidis et al., 2018). More recently, its application in energy systems has attracted considerable attention due to its capacity to improve efficiency, reliability, and adaptability (Wang et al., 2018).

Energy systems encompass a diverse range of entities, including individual devices, buildings, industrial facilities, and large-scale infrastructure such as smart grids. Effective management of these systems requires intelligent control mechanisms to maintain operational stability, respond to fluctuating inputs (e.g., variations in demand), and optimize energy flows. For example, short-term forecasting of electricity demand and renewable energy generation can facilitate more efficient scheduling of conventional power plants, thereby reducing fuel consumption and emissions. Such predictive capabilities require dynamic, data-driven models with integrated decision-making functions, which cannot be achieved using traditional static methods. AI-based systems, particularly those employing ML techniques, enable continuous learning from both historical and real-time data, thereby improving operational accuracy and supporting autonomous system control. A key application of AI and ML is in modelling building energy consumption (Agustín and López, 2009; Banos et al., 2011). Predicting energy usage in building is inherently complex due to the interaction of multiple factors, including structural properties, weather conditions, equipment performance, and occupant behaviour (Fazlollahi et al., 2012). Techniques such as support vector machines (SVM), artificial neural networks (ANN), and decision tree methods have been extensively applied to address these challenges (Fazlollahi et al., 2015). Furthermore, optimizing of heating, ventilation, and air conditioning (HVAC) systems, as well as absorption chillers, using methods such as genetic algorithms (GA) and ANN, has demonstrated substantial improvement in both energy efficiency and thermal comfort (Evins, 2015; Huang et al., 2019).

In industrial applications, particularly within the chemical sector, AI and ML have demonstrated considerable potential for process optimization and control (Bahrami and Amini, 2018). Examples include combustion modelling (Narayan and Ponnambalam, 2017), optimization of distillation columns (Evins, 2013), and specialized processes such as heavy oil fractionation (Chen and Yang, 2018). These approaches contribute to specialized process efficiency, reduced energy consumption, and improved operational stability. More broadly, ML techniques identify patterns within datasets to support decision-making, while ANN models provide robust tool for modelling nonlinear relationship in complex systems. AI applications also extend to infrastructure systems such as water distribution networks, where ANN-based models have been employed to simulate hybrid systems and improve energy efficiency in both production and operational processes (Kim et al., 2016; Miao et al., 2011). In the renewable energy domain, AI and ML have become increasingly important for forecasting and optimizing task. Photovoltaic (PV) systems, for instance benefit from predictive models that integrate historical performance data with weather forecast to enhance power generation and system design (Halder et al., 2017; Halder and Samad, 2017; Kadhim and Rona, 2018a; Thomsen et al., 2018). Similarly, wind energy systems have been extensively analysed using ML-based forecasting techniques to improve generation efficiency (Kadhim and Rona, 2018b). Moreover, AI-driven methodologies are gaining prominence in emerging area such as energy management for electric vehicles and renewable-integrated grid systems. Although conventional physics-based models remain valuable for long-term projections, the often lack the responsiveness requires for short-term forecasting. In contrast, AI-based models-including ANN and fuzzy logic systems-offer greater adaptability and improved predictive performance in dynamic operating environments (Sanchez and Martín, 2018). Artificial intelligence and machine learning approaches differ in their data requirements, interpretability, and suitability for energy materials and energy system applications. Artificial neural networks (ANNs) are widely used when nonlinear relationships exist between input variables and target outputs. Typical inputs include experimental parameters, operating conditions, structural descriptors, material composition, environmental variables, and historical performance data. ANNs are particularly useful for predicting energy consumption, battery performance, photovoltaic output, electrolyte behavior, and process optimization. Their key advantage is their ability to model complex nonlinear relationships; however, they generally require large datasets, careful architecture selection, and are often criticized for limited interpretability. Support vector machines/support vector regression (SVM/SVR) are effective for classification and regression problems, especially when datasets are small to medium in size. Inputs may include physiochemical description, process variables, spectral features, or energy system parameters. SVM based models are useful for forecasting, faulty detection, energy consumption prediction, and materials screening. Their advantages include strong performance with limited datasets and robustness in high dimensional features spaces. However, their performance depends heavily on kernel selection and parameter tuning, and they may become computationally expensive for very large datasets. Surrogate models, also known as meta models, are simplified data driven approximations of computationally expensive physics based or engineering models Their inputs are usually design variables, operating parameters, techno-economic parameters, and simulation outputs from actual engineering models. They are useful in optimization problems, and simulation outputs from actual engineering models. They are useful in optimization problems where repeated simulations are computationally expensive. Perera et al. developed an ANN based surrogate model for distributed energy system optimization and combined it with an actual engineering model to reduce computational burden while maintaining accuracy. The study reported that the hybrid optimization algorithm reached Pareto solutions approximately 17 times faster than the actual engineering model and reduced computational time by up to 84%.

## 4 AI-driven approaches for energy-efficient manufacturing systems

While the bibliometric analysis discussed in the previous section identifies key research trends and thematic focuses in AI-enabled manufacturing, the current section provides a qualitative review of existing literature to analyze the principal challenges and corresponding solution strategies proposed in earlier studies. The first dimension addresses the primary energy-efficiency challenges in manufacturing systems, namely,: (1) monitoring and prediction, (2) real-time control, (3) scheduling, and (4) parameter optimization. The second dimension examines the role of artificial intelligence within these approaches, distinguishing between studies where AI serves as the central solution framework and those where it functions as a supporting or enabling component. The third dimension classifies the methodological structure of the proposed solution, including machine learning (ML), neural networks (NN), deep learning (DL), and reinforcement learning (RL). The following subsections analyze data-driven approaches reported in the literature for addressing the identified energy-efficiency challenges in manufacturing systems. The associated tables compile key algorithm details and are structured according to the following criteria:

### 4.1 Objective

This denotes the intended energy-related outcome of the proposed approach, such as reducing energy consumption, lowering energy costs, or enhancing overall energy performance.

### 4.2 Input and output

These categories describe the data utilized by the algorithms and the corresponding outputs they produce. In RL-based methods, or approaches incorporating RL, inputs are typically represented as state variables, while outputs correspond to actions. More generally, data used in AI applications can be classified into two groups: manufacturing-related and non-manufacturing-related data. Manufacturing data may be directly acquired from production systems at different hierarchical levels-such as component, machine, or production line or derived from external sources linked to manufacturing operations. Component-level data include information related to machine parts or tooling operations. Component-level data encompass parameters such as cutting speed, feed rate, and spindle dynamics. Line-level data generally include equipment status, operating conditions, and process variables such as temperature and pressure. Additionally, inputs may involve job-specific information, including operation sequences, electricity market data such as pricing, weather-related parameters such as ambient temperature and temporal indicators such as calendar effects. Non-manufacturing data refer to cases where AI is not primary solution approach but is instead used to enhance or support an existing analytical or optimization framework.

### 4.3 Testbed

This criterion reflects the level of validation of the proposed method under realistic conditions. “Simulation” indicates validation in a virtual environment or using synthetically generated datasets. “Experimental data” refers to testing conducted with real-world data, though not under liver operational conditions. “Real-world” signifies validation within an actual industrial setting.

### 4.4 Controlling variables

These represent the decision variables through which the energy performance can be influenced. Such variables are particularly relevant in problem domains involving decision-making, including scheduling, real-time control, and parameter optimization. Similar to input data, controlling variables may be categorized as manufacturing related to non-manufacturing related. Manufacturing-related variables can exist at the component, machine, or production-line level. Component-level variables pertain to specific machine elements, machine-level variables include operational parameters such as cutting speed and feed rate, and line-level variables involve production status and process conditions. Non-manufacturing control variables correspond to algorithmic or methodological configurations applied when AI serves as a supplementary rather than primary solution mechanism.

This framework facilitates a synthetic evaluation of how AI-based methods have been developed and applied to enhance energy efficiency in manufacturing, while also clarifying their scope, data requirements, and level of implementation maturity.

### 4.5 Monitoring and prediction

Monitoring and prediction from a fundamental layer in achieving energy efficiency within manufacturing systems, supporting higher-level functions such as scheduling, real-time control, and parameter optimization. Existing studies address these aspects across multiple system levels, including individual machines and entire production lines. This domain encompasses several key subproblems, such as energy consumption prediction, pattern recognition, remaining useful life estimation, fault diagnosis, and analysis of human-machine interactions. These subproblems can be broadly classified into two categories: energy analytics, which focuses on predicting energy consumption and identifying usage patterns, and predictive monitoring, which includes remaining useful life estimation, fault detection, and interaction-aware monitoring systems. In general, the proposed methodologies emphasize data-driven analysis rather than direct decision-making and predominantly reply on artificial intelligence techniques, including classical machine learning methods, shallow neural networks, and deep learning architectures. Among these, deep learning models particularly long short-term memory (LSTM) networks are most widely utilized due to their capability to capture temporal dependencies in sequential datasets. Input data typically consist of multi-level manufacturing information, supplemented by external variables such as electricity prices and time-dependent features.

#### 4.5.1 Energy analytics

**FIGURE 2:**
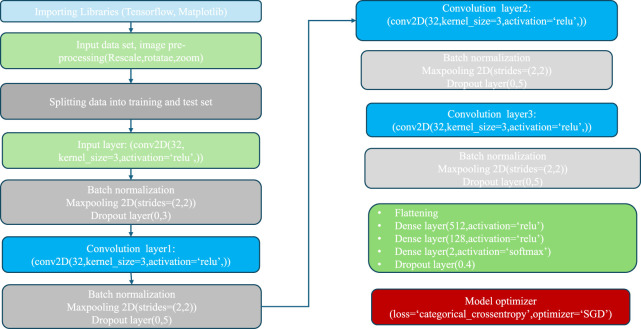
Structure of CNN code employed in a study.

The main objective of energy analytics is to develop predictive models for estimating energy consumption and identifying usage patterns within manufacturing environments. A substantial portion of the literature focuses on dep neural network architectures, particularly LSTM and convolutional neural networks (CNN), for these applications (Figure 2). LSTM-based models, often enhanced with optimization techniques such as particle swarm optimization (PSO), have been employed to predict energy consumption in machining processes. Similarly, integrated framework combining LSTM with computer vision methods-such as object detection and human pose estimation-have been used to incorporate human-machine interaction effects in complex production systems. To further improve predictive performance, hybrid architectures that integrate spatial and temporal learning mechanisms have been proposed. These include combinations of convolutional networks or graph-based models with gated recurrent units (GRUs), enabling simultaneous modelling of spatial relationships and temporal dynamics across interconnected production systems. Additionally, clustering-based frameworks that combine and analyze energy consumption patterns in industrial settings. Comparative analyses have also assessed the performance of deep learning models-such as CNNs, stacked autoencoders, and deep belief networks-relative to classical machine learning methods under varying operational conditions, including tool wear equipment degradation.

**FIGURE 3:**
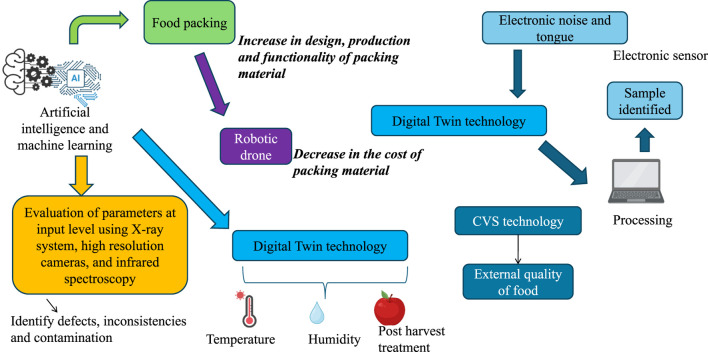
Role of Artificial intelligence and Machine learning in the food industry.

Shallow neural network models have also been investigated for energy modelling and pattern recognition. These include feedback neural networks with varying depths and neuron configurations, optimized using techniques such as backpropagation and evolutionary algorithms. Applications range from identifying key determinants of energy consumption in CNC machining to modelling energy usage in processes such as laser welding and injection moulding. In certain cases, hybrid models combining neural networks with clustering methods, such as K-means, have been proposed to generate representative energy consumption scenarios. Beyond neural network-based approaches, several studies have explored classical machine learning techniques for modelling energy consumption. Algorithms such as random forests (RF), decision trees, Gaussian process regression (GRP), and polynomial or linear regression have been applied across diverse manufacturing contact. For example, RF models have demonstrated higher predictive accuracy compared to decision tree method in machining operations, while SRP has been utilized for modelling energy consumption in machine tools due to its flexibility as a nonparametric approach. Other hybrid strategies combine multiple methodologies such as kernel-based learning, Petri net modelling, and digital twin frameworks to represent complex systems behaviours (Figure 3). Clustering techniques, including fuzzy c-means, have also been applied to characterize process complexity and its relationship with energy consumption, particularly in advance manufacturing environments.

#### 4.5.2 Predictive monitoring

**FIGURE 4:**
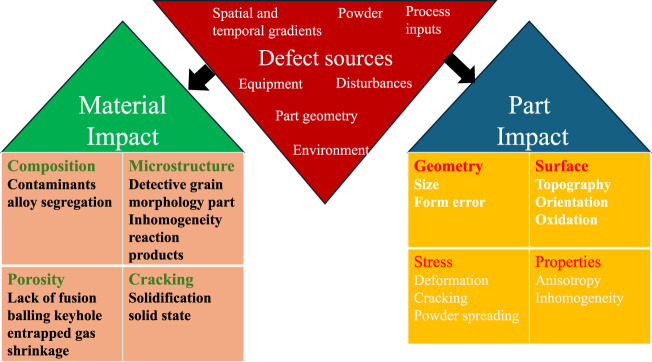
Classification of defects in metal AM.

Predictive monitoring focuses on developing models capable of estimating the remaining useful life of equipment, detecting faults and anomalies, and analyzing human-machine interactions. These capabilities care critical for enhancing system reliability, reducing unplanned downtime, and improving energy efficiency through proactive maintenance strategies (Figure 4). Artificial intelligence techniques-particularly neural networks and deep learning architecture are extensively used for these purposes. Recurrent models such as long short-term memory (LSTM) networks have been effectively applied for remaining useful life prediction in applications including robotic batter systems and CNC machining processes under varying operating conditions (Bagga et al., 2022; Mallouk et al., 2023). More advanced hybrid approaches incorporate attention mechanisms and optimization techniques, for example, bidirectional LSTM models enhance with fuzzy attention and metaheuristic optimization algorithms have been utilized for fault diagnosis in rotating machinery (Alassery and Alhazmi, 2022). These methods are often combined with signal processing techniques such as empirical mode decomposition for feature extraction, along with dimensionality reduction methods such as principal component analysis to improve predictive performance. In addition to fault diagnosis, defect detection has also been significantly enhanced through deep learning techniques. Architectures such as neural networks and object detection frameworks (e.g., YOLO-based models) have been employed for feature extraction, localization, and classification of defects in manufacturing components, including metal gear surfaces (Su et al., 2022). Alongside deep learning, traditional unsupervised machine learning methods remain relevant, particularly for anomaly detection tasks. Clustering techniques such as K-means have been applied to identify abnormal energy consumption patterns and operational inefficiencies (Mendia et al., 2024). Notably, these approaches emphasis interpretability, enabling operators and decision-makers to better understand and trust the outputs of AI-driven systems-an essential consideration for practical industrial implementation.

### 4.6 Real-time control

Energy-efficient real-time control in manufacturing systems involves dynamically regulating machines or production lines within operational time consytraints to achieve energy-related objectives. This includes actions such as minimizing idle durations, adjusting operational states, and coordinating system components to optimize energy utilization. The primary objectives addressed in the literature include reducing energy consumption and minimizing energy costs, leading to two main subclasses: (i) energy consumption-based control and (ii) energy price-based control. Across both categories, deep reinforcement learning (DRL) techniques have emerged as dominant approaches, particularly algorithms such as deep Q-networks (DQN) and actor-critic (AC) methods. These techniques are typically employed as primary decision-making tools, utilizing system-level data such as production line conditions, electricity pricing and environmental variables. Control variables are predominantly defined at the production line level.

#### 4.6.1 Real-time energy consumption based control

**FIGURE 5:**
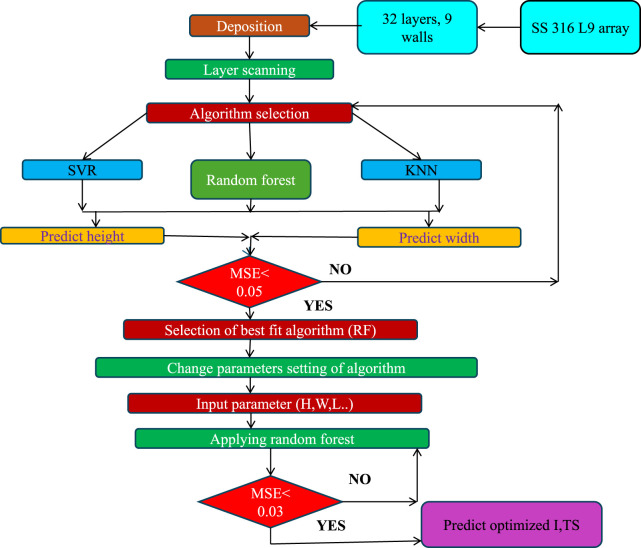
ML models (RF, KNN, and SVR) were run offline to obtain starting values for current and travel speed and an inverse RF model was run online in real-time (based on measured layer dimensions) for the in-situ continuous optimization of current and travel speed.

In this category, the objective is to regulate manufacturing processes in real time based on energy consumption patterns. Several studies propose AI-driven control strategies using reinforcement learning techniques. For example, actor-critic methods incorporating radial basis function neural networks have been applied to control hybrid manufacturing systems characterized by both discrete and continuous dynamics (Schwung et al., 2019). Similarly, DQN-based methods have been utilized to derive optimal control policies for single workstation with parallel machines, while proximal policy optimization (PPO) approaches have demonstrated improved performance in more complex multi-stage production systems (Loffredo et al., 2023a; Loffredo et al., 2023b). In addition to fully AI-driven approaches, hybrid methodologies where AI functions as a supporting component have also been proposed. These include frameworks that integrate machine learning models such as Gaussian mixture models, neural networks, or support vector regression-with optimization algorithms to guide decision-making. For instance, predictive models of machine idle time can be incorporated into simulation environments or optimization frameworks such as NSGA-II to inform energy control strategies (Gao et al., 2023; Zhang et al., 2021). Other approaches combine predictive modelling with genetic algorithms to optimize parameters of HVAC systems or manufacturing equipment (Wang et al., 2021). Statistically learning techniques, including kernel density estimation and likelihood-based methods, have also been applied to model system uncertainties and support optimization processes (Frigeri et al., 2021; Frigerio et al., 2021). Additionally, classification methods such as k-nearest neighbours (KNN) have been integrated into intelligent process management systems to enhance decision-making in manufacturing operations (Jo et al., 2020) (Figure 5).

#### 4.6.2 Real time energy price-based control

In contrast, energy price-based control aims to minimize operational cost by adjusting system behaviour in response to dynamic electricity pricing signals. Multi-agent reinforcement learning frameworks particularly those based on actor-critic methods and PPO-have been applied to flexible manufacturing systems operating under uncertain conditions, such as fluctuating electricity prices influenced by weather variations (Bakakeu et al., 2021). In these frameworks, individual agents control specific system components, enabling coordinated and adaptive decision-making. DQN-based approaches are also widely employed in this context. Variants such as decomposed multi-agent DQN improve interpretability by distributing control responsibilities among multiple agents while enhancing overall performance compared to conventional DQN models (Yun et al., 2023). Similarly, double DQN techniques have been utilized to mitigate overestimation issues in value functions, particularly in systems involving energy storage and grid interactions (Yi et al., 2022). Hybrid approaches, where AI supports traditional optimization techniques, are less frequently reported but remain relevant. For example, optimization techniques, are less frequently reported but remain relevant. For example, predictive models based on LSTM networks have been used to generate forecast guide mathematical optimization solvers in quantifying cost-effective control strategies (Lu et al., 2021).

## 5 A conceptual Energy-Efficient Digital Twin (EE-DT) framework

In recent years, digital twin (DT) technology has emerged as a fundamental component of intelligent manufacturing, primarily due to its ability to improve operational efficiency and facilitate data-driven decision-making (Liu et al., 2024; Yin et al., 2024). Fundamentally, a DT represents a high-fidelity digital replica of a physical manufacturing system including machines, tools, and process elements, that can interact with its physical counterpart in a bidirectional and real-time manner (Xie et al., 2024). This capability enables continuous monitoring, evaluation, and optimization of system performance. From an architectural standpoint, the DT serves as an intermediate layer connecting the physical and digital domains. On one side, it organizes and interprets raw data acquired from the physical environment, enabling precise representation, visualization, and analysis of system states within the digital domain (Pandhare et al., 2024). On the other side, it converts analytically derived or AI-enabled control strategies into executable commands that can be deployed within the physical systems, thereby bridging the divide between digital intelligence and real-world implementation (Zhou et al., 2024). The DT framework generally comprises three core components: information modelling, communication infrastructure, and data processing. Standardised information models are employed to describe and represent physical assets, while communication networks ensure continuous synchronization between physical and digital entities through ongoing data exchange. Data processing central to this work encompasses data pre-processing, monitoring and prediction, and decision-making. Pre-processing activities include data cleaning, storage, and stream management to ensure low-latency and high-quality data availability. Monitoring and prediction from the analytical core that supports subsequent decision-making processes.

DT technology has been extensively applied across various areas of energy-efficient manufacturing. Previous studies have utilized DT for energy modelling (Liu et al., 2023), optimization pf process and machining parameter (Pantelidakis and Mykoniatis, 2024). Scheduling optimization and real-time control based on energy consumption as well as energy pricing. Despite these advancements, the incorporation of AI-driven methodologies within DT frameworks remains relatively limited, particularly in decision-making applications. Most existing research has concentrated on monitoring and prediction task (Abdoune et al., 2023), leaving a notable gap in the application of DT for real-time control, scheduling and optimization. To address this limitation, a comprehensive conceptual framework termed the Energy-Efficient Digital Twin (EE-DT) is introduced. This framework integrates AI techniques with DT architecture to systematically tackle energy efficiency challenges in manufacturing systems. The EE-DT framework comprises two primary layers: the physical layer, representing the actual manufacturing environment, and the digital layer, where data processing, analytics, and decision-making are performed. Data exchange between these layers occurs bidirectionally; informational data (e.g., system status and performance indicators) are transmitted from the physical system to the digital layer, while control signals and optimization outputs are communicated back to the physical system.

Machine learning (ML) integrated techno-economic analysis (TEA) has emerged as a transformative strategy for evaluating and optimizing biofuel production systems (Anto et al., 2021; Balaji et al., 2021). The integration of ML into TEA enables the incorporation of multidisciplinary datasets derived from algal biorefinery engineering, catalytic conversion technologies, wastewater remediation systems, environmental monitoring, advanced materials science, and sustainability assessment. Such an integrated framework supports improved prediction accuracy, reduced uncertainty, and enhanced decision-making capabilities for large-scale implementation of sustainable biofuel technologies. Microalgae are increasingly recognized as an efficient and environmentally sustainable feedstock for next-generation biofuel production because of their rapid growth kinetics, elevated carbon fixation capacity, and ability to proliferate in non-arable and wastewater-based cultivation systems (Balaji et al., 2022; Cha et al., 2024; Chang et al., 2025; Chi et al., 2021). These characteristics significantly reduce dependency on freshwater resources and agricultural land while simultaneously supporting environmental remediation and resource recovery. The integration of wastewater treatment with algal biomass generation further improves the economic and ecological feasibility of algal biorefineries by coupling nutrient recovery with bioenergy production. Recent developments in algal biorefinery engineering have demonstrated that optimization of polysaccharide utilization pathways, biomass conversion strategies, and AI-assisted operational control can substantially improve overall process efficiency and techno-economic viability. ML-assisted frameworks facilitate the evaluation of process variables such as nutrient concentration, cultivation conditions, harvesting efficiency, lipid productivity, and downstream conversion performance. Consequently, these computational approaches support the identification of economically favorable and environmentally sustainable operational configurations. In parallel, substantial advances in engineered nanomaterials and catalytic systems have contributed to enhanced environmental performance and energy conversion efficiency in microalgal processing systems (Datta et al., 2020; Datta et al., 2021; Ethiraj et al., 2024; Ganesan et al., 2020; Maheshwaran et al., 2022a). Carbon-based nanostructures, graphene derivatives, metal organic frameworks (MOFs), and multifunctional hybrid composites exhibit excellent adsorption properties, catalytic activity, and physicochemical stability. The incorporation of these materials into algal biofuel production systems improves pollutant removal, enhances catalytic conversion pathways, and supports efficient biomass processing. In particular, graphene derived materials possess high surface area, tunable surface chemistry, and superior electrical conductivity, making them highly effective for adsorption and catalytic applications in aqueous environments.

Similarly, biochar-derived catalysts and porous hybrid materials have gained considerable attention for their role in biofuel production and environmental remediation (Maheshwaran et al., 2022b; Maheshwaran et al., 2022c). Their enhanced catalytic properties contribute to improved reaction kinetics, increased conversion efficiency, and optimized energy generation pathways. The incorporation of these advanced functional materials into ML-assisted TEA models enables improved assessment of process sustainability, material efficiency, and lifecycle performance. Wastewater integrated algal cultivation systems are often exposed to heavy metals, emerging contaminants, organic pollutants, and toxin accumulation, creating additional operational and environmental challenges (Maheshwaran et al., 2022d; Nagendran et al., 2024; Natishah et al., 2025; Punna et al., 2026). Extensive investigations on adsorption mechanisms, bioremediation pathways, advanced oxidation technologies, and pollutant degradation systems provide valuable datasets that can be integrated into ML models for realistic techno-economic evaluation. These datasets support predictive modeling of contaminant behavior, operational variability, treatment efficiency, and environmental impact under large-scale conditions. Furthermore, enzyme immobilization technologies and microbial catalytic systems have shown substantial potential for improving pollutant degradation and biomass conversion efficiency (Pusuluru et al., 2026a; Pusuluru et al., 2026b; Pusuluru et al., 2026c). Immobilized enzymatic systems enhance process stability, catalytic efficiency, and substrate specificity while simultaneously reducing operational costs associated with enzyme recovery and reuse. The integration of these biochemical and catalytic datasets into ML-assisted frameworks significantly improves the robustness and scalability of TEA-based optimization strategies. Growing concern regarding persistent and emerging contaminants, particularly per- and polyfluoroalkyl substances (PFAS), has further emphasized the importance of integrating environmental risk assessment and regulatory considerations into biofuel techno economic frameworks (Rajnish et al., 2021; Samuel and Chidambaram, 2015). The transformation, persistence, transport, and potential toxicity of such contaminants during thermal, catalytic, and electrochemical treatment processes must be carefully considered to ensure environmental safety and long-term sustainability. In addition, groundwater contamination, trace element mobility, and variations in water quality highlight the need to expand TEA boundaries beyond traditional economic indicators to include lifecycle sustainability and ecological risk management. Advances in environmental sensing technologies have also accelerated the integration of ML within TEA frameworks (Samuel et al., 2018; Samuel et al., 2021; Samuel et al., 2022a; Samuel et al., 2022b; Samuel et al., 2023). Electrochemical sensors, nanomaterial-assisted sensing systems, and surface-enhanced spectroscopic platforms provide rapid and sensitive monitoring of contaminants, reaction intermediates, and process parameters. Real-time data acquisition from these analytical systems enables adaptive process control and dynamic optimization of operational variables. The incorporation of such continuous monitoring data into ML algorithms enhances prediction accuracy, improves process stability, and supports intelligent decision-making in integrated bioenergy systems.

Recent progress in heterojunction nanocomposites, electrochemical sensing materials, and hybrid catalytic architecture has further improved the selectivity, sensitivity, and response performance of environmental monitoring systems. These technologies provide critical mechanistic insights into catalytic behavior, adsorption pathways, and degradation efficiency, thereby strengthening the reliability of ML-assisted optimization models. From a sustainability perspective, circular material systems represent an important advancement for future algal biorefineries (Samuel et al., 2024; Sekhar et al., 2024; Selvarajan et al., 2025). The conversion of waste plastics into functional carbon nanomaterials, the synthesis of bio-derived graphene, and the development of smart polymeric systems for water treatment contribute significantly to circular economy strategies and waste valorization approaches. Such technologies minimize environmental burden while simultaneously generating value-added materials for energy and environmental applications. In addition, advanced nano-catalysts and enzyme-assisted catalytic systems have demonstrated substantial potential for improving CO2 conversion, enhancing bioenergy generation, and promoting carbon-neutral energy pathways (Senthilkumar et al., 2024; Singh et al., 2024a; Singh et al., 2024b; Singh et al., 2025). The integration of ammonia synthesis technologies, hydrogen economy concepts, and sustainable catalytic systems into bioenergy platforms further highlights the potential for coupling microalgal biofuel systems with emerging renewable energy infrastructures. The application of advanced nanocomposites also extends to corrosion-resistant coatings, functional electrode materials, and hybrid catalytic surfaces, which collectively improve system durability, operational efficiency, and material stability under harsh environmental conditions (Sivasubramanian et al., 2024; Steffi et al., 2022a; Steffi et al., 2022b). These innovations, when integrated with ML-driven predictive optimization and TEA frameworks, support the development of adaptive, scalable, and economically viable bioenergy systems. Overall, ML-assisted TEA frameworks represent a major advancement in the optimization and commercialization of microalgal biofuel technologies. By integrating multidisciplinary datasets related to environmental conditions, material properties, catalytic behavior, sensing technologies, process dynamics, and sustainability indicators, ML-based approaches provide a comprehensive platform for evaluating system performance and minimizing operational uncertainty. Such holistic frameworks are essential for accelerating industrial-scale implementation of microalgal biofuel systems while ensuring environmental compatibility, regulatory compliance, and long-term economic sustainability.

### 5.1 Energy-focused digital twin architecture

The energy-oriented DT component within the EE-DT framework is organized into five key modules: monitoring and Prediction, Real-Time Control, Scheduling, Parameter Optimization, and Energy Efficiency Objectives. The first four modules correspond to the principal energy-related challenges in manufacturing systems, whereas the fifth defines the optimization objectives that guide system performance. These objectives may include indicators such as energy consumption (E), energy cost (EC), carbon emission (CE), make span (C), productivity (P), product quality (PQ), maintenance (M), flow time (F), lateness (L), tardiness (T), earliness (EA), and waiting time (W). Within this architecture, input data are initially processed in the monitoring and prediction module, which provides the analytical basis for higher-lever decision-making functions. The insights generated are subsequently integrated with predefined energy efficiency objectives to guide decision-making processes in the real-time control, scheduling, and control strategies, and parameter configurations are the implemented within the physical system through feedback mechanism.

Each energy-efficient module is further divided into three subcomponents:

- Sub-problems: Specific issues associated with each energy-related domain.
- AI approaches: Relevant machine learning or optimization techniques
- Application layer: Practical implementation pathways linking AI methods to real-world solutions

This structured design supports the systematic integration of AI techniques into manufacturing operations, enabling scalable and adaptive energy management.

## 6 Research challenges

Although the proposed EE-DT framework establishes a comprehensive basis for integrating AI into energy-efficient manufacturing, several important challenges persist. These include defining appropriate energy efficiency objectives, which requires balancing multiple and often conflicting criteria such as cost, emissions, and productivity; identifying energy-saving opportunities though accurate detection of inefficiencies in complex and dynamic systems; addressing challenges related to energy data, including issues of data quality, availability, and integration across heterogenous source; and implementing AI solutions in real-world environments while ensuring robustness, scalability, interpretability, and seamless compatibility with existing industrial infrastructures. Addressing these issues is crucial for enabling the practical deployment of AI-enabled DT frameworks and fully realizing their potential in sustainable manufacturing systems.

## Data Availability

The original contributions presented in the study are included in the article/supplementary material, further inquiries can be directed to the corresponding authors.
